# The Analgesic Potential of Litsea Species: A Systematic Review

**DOI:** 10.3390/molecules29092079

**Published:** 2024-04-30

**Authors:** May Poh Yik Goh, Raudhatun Na’emah Samsul, Amal Widaad Mohaimin, Hui Poh Goh, Nurul Hazlina Zaini, Nurolaini Kifli, Norhayati Ahmad

**Affiliations:** 1 Herbal Research Group, Universiti Brunei Darussalam, Jalan Tungku Link, Gadong, Bandar Seri Begawan BE 1410, Brunei; may.goh@ubd.edu.bn (M.P.Y.G.); raudhatunhs@gmail.com (R.N.S.); amal.mohaimin@ubd.edu.bn (A.W.M.); nurolaini.kifli@ubd.edu.bn (N.K.); 2 PAP Rashidah Saádatul Bolkiah Institute of Health Sciences, Universiti Brunei Darussalam, Jalan Tungku Link, Gadong, Bandar Seri Begawan BE 1410, Brunei; pohhui.goh@ubd.edu.bn; 3 Environmental and Life Sciences, Faculty of Science, Universiti Brunei Darussalam, Jalan Tungku Link, Gadong, Bandar Seri Begawan BE 1410, Brunei; 4 UBD Botanical Research Centre, Institute for Biodiversity and Environmental Research, Universiti Brunei Darussalam, Jalan Tungku Link, Gadong, Bandar Seri Begawan BE 1410, Brunei; hazlina.zaini@ubd.edu.bn

**Keywords:** *Litsea*, Lauraceae, analgesic, antinociception, pain management, pain, acetic acid, tail flick, inflammatory, 5-hydroxytryptamine 1A

## Abstract

Various plant species from the *Litsea* genus have been claimed to be beneficial for pain relief. The PRISMA approach was adopted to identify studies that reported analgesic properties of plants from the *Litsea* genus. Out of 450 records returned, 19 primary studies revealed the analgesic potential of nine *Litsea* species including (1) *Litsea cubeba*, (2) *Litsea elliptibacea*, (3) *Litsea japonica*, (4) *Litsea glutinosa*, (5) *Litsea glaucescens*, (6) *Litsea guatemalensis*, (7) *Litsea lancifolia*, (8) *Litsea liyuyingi* and (9) *Litsea monopetala*. Six of the species, 1, 3, 4, 7, 8 and 9, demonstrated peripheral antinociceptive properties as they inhibited acetic-acid-induced writhing in animal models. Species 1, 3, 4, 8 and 9 further showed effects via the central analgesic route at the spinal level by increasing the latencies of heat stimulated-nocifensive responses in the tail flick assay. The hot plate assay also revealed the efficacies of 4 and 9 at the supraspinal level. Species 6 was reported to ameliorate hyperalgesia induced via partial sciatic nerve ligation (PSNL). The antinociceptive effects of 1 and 3 were attributed to the regulatory effects of their bioactive compounds on inflammatory mediators. As for 2 and 5, their analgesic effect may be a result of their activity with the 5-hydroxytryptamine 1A receptor (5-HT_1A_R) which disrupted the pain-stimulating actions of 5-HT. Antinociceptive activities were documented for various major compounds of the *Litsea* plants. Overall, the findings suggested *Litsea* species as good sources of antinociceptive compounds that can be further developed to complement or substitute prescription drugs for pain management.

## 1. Introduction

Pain has been classified as a major public health concern where it has affected approximately 20% of adults globally, with around 1 in 10 adults diagnosed with chronic pain each year [1]. As one of the top contributors to the global burden of diseases, pain has become one of the most expensive conditions, incurring over $134.5 billion for only lower back and neck pain in 2016 in the USA [2]. Pain can arise from various medical conditions including, but not limited to, osteo- and rheumatoid arthritis, cancer, spinal problems, operations and injuries; thus, the pathophysiology of pain can be complex and challenging to deduce [1]. Nevertheless, the neurophysiological bases and molecular mechanisms for the etiology of chronic pain such as neuronal plasticity, microglial and astrocyte activation and immune-cell infiltration have been well-documented and proposed [3,4]. 

The pathophysiological changes involved in pain development or attenuation can be modulated by modifications in various endogenous peptidergic pathways, including kinins, calcitonin gene-related peptide (CGRP), growth factors, opioids and somatostatin, and non-peptidergic pathways such as catecholamines, histamine, purine nucleosides, prostaglandins, excitatory amino acids and cannabinoids [4]. As a form of pharmacological intervention, opioids and nonsteroidal anti-inflammatory drugs (NSAIDs) have been prescribed as standard therapies for pain management. Opioids such as heroin and morphine, as well as endorphins, an endogenous peptide ligand, can target the opioid family of receptors, whereas NSAIDs, like aspirin and indomethacin, can inhibit cyclooxygenase-1 (COX-1) and cyclooxygenase-2 (COX-2) enzymes which are associated with the synthesis and release of prostaglandins and CGRP [5]. Unfortunately, the use of opioids and NSAIDs, either short- or long-term, can result in moderate to adverse side effects including nausea, vomiting, drowsiness, weakness, constipation, urinary retention, sedation, mild cognitive impairment, myoclonus, respiratory depression, opioid-induced hyperalgesia, diminished bone density, persistent bleeding, myocardial infarctions and strokes, as well as gastrointestinal complications, such as dyspepsia, gastric ulcers, erosion and mucosal hemorrhage and other musculoskeletal and renal conditions [6,7,8,9]. As a consequence, the search for effective alternatives that can bypass the detrimental side effects of existing analgesic medications still persists [10,11,12,13,14,15,16]. 

Natural products have been evidently shown as potential alternative therapies to conventional treatments in view of their nominal side effects while also offering a cost-effective source for bioactive ingredients that can potentially be developed into new drugs and remedies of high efficacy [17,18]. FDA-approved analgesics such as ziconotide, capsaicin and botulinum toxin type A have presented novel mechanisms for their analgesic activity [19]. Investigations on other compounds such as *Conus* venom peptides (MrIA, Contulakin G, Vc1.1, conantokin G), capsaicinoids, capsinoids and their synthetic analogues are also underway for development into new analgesics [19]. Despite these advances, progressive pharmacological investigations have continued to prospect other plant species as potential new sources of analgesic compounds including *Litsea* genus from the Lauraceae family [20,21,22,23,24,25,26].

The laurel family, Lauraceae, encompasses about 55 genera and 4000 species of aromatic trees or shrubs, most of which can be found in the warm tropics of Southeast Asia and South America [27]. *Litsea* represents an ecological and economical vital genus of the Lauraceae family that includes roughly 400 species, which are typically recognized as evergreen/deciduous trees or shrubs with alternating leaves that are rarely opposite or verticillate and umbel inflorescence consisting of four-celled introrse anthers and an involucre of persistent, alternate and opposite bracts [28]. Most *Litsea* species are distributed in tropical and subtropical Asia, although few *Litsea* species have also been reported in Australia, the Pacific islands and North to South America, as well as other temperate regions [28,29,30]. In traditional and indigenous medicine, the leaves and bark of the *Litsea* species have broad applications such as for the treatment of pain, headache, fever, stomachaches, inflammation, diarrhea, vomiting, dyspepsia, gastroenteritis, diabetes, edema, arthritis, asthma, intoxication, seminal weakness and central nervous disorders [27,31,32,33,34]. Based on folkloric records, more than twenty *Litsea* species have been claimed as useful remedies for pain management. Table 1 lists the plants of the *Litsea* genus that have been reported to have traditional uses against pain along with their other ethnomedicinal uses, specific applications and reported phytochemicals for each of the plant species. These beliefs served as an important basis for scientific evaluations to substantiate the pharmacological values of these traditional medicinal plants.

In vitro and in vivo investigation on the crude extracts, fractions and phytochemical content revealed that various *Litsea* species exhibited analgesic, anti-inflammatory, antibacterial, antioxidant, antidiabetic, anti-diarrheal, anti-fungal, anti-arrhythmic, anti-HIV, anticancer, insecticidal, wound healing, antidepressant, cardioprotective and cytotoxic activities [35,36]. The diverse pharmacological benefits of the *Litsea* species have been attributed to their rich composition of alkaloids, flavonoids, terpenoids, saponins, tannins, lactones and volatile oils [35,37]. In particular, 1,8-cineole (eucalyptol) has been identified as a major component of the *Litsea* species followed by tetrahydrolinalool, limonene, methyl eugenol and dihydrocarvone [38]. According to Pérez et al. (2011) [38], 1,8-cineole-rich species are popular as spices, while those with abundant linalool and limonene are generally used in traditional medicine. Nevertheless, 1,8-cineole has been reported as a novel antagonist of human transient receptor potential ankyrin 1 (TRPA1) and has potent analgesic and anti-inflammatory effects [39]. Furthermore, the other two major *Litsea* constituents, linalool and limonene, have also exhibited notable antinociceptive activity [40,41], suggesting the high propensity for the plants of the *Litsea* genus as potential sources of natural analgesics. Indeed, the analgesic capacity of numerous *Litsea* species has been unveiled in a number of in vitro, in vivo and clinical studies. This review aims to compile and summarize the different *Litsea* species that have been reported to have analgesic effects and the potential phytoconstituents responsible for the antinociceptive action.

**Table 1:** Ethnomedicinal claims and phytochemicals reported for *Litsea* species with proclaimed pain-relieving properties.

Species/Vernacular Name	Ethnomedicinal Claims	Plant Parts Used	Preparation/Application	Phytochemical Reported	References
*Litsea cubeba* (Lour.) Pers. Mountain Spicy Tree; *Tayer* (Indian); *Cheng Qie Zi*/*Shan Ji Jiao* (Chinese); *Siltimur* (Nepali)	Analgesic, anti-asthmatic, anti-dysenteric, anti-inflammatory, antiseptic, astringent, carminative, diuretic, expectorant, hair tonic, hypotensive, insecticidal, sedative, stimulant, stomachic, warms body core, moves ‘*qi*’ and improves blood circulation. Relieves pains including headache, toothache, stomachache and athlete’s foot pain, cough, cold, respiratory diseases, chronic bronchitis, asthma, cholera, food accumulation, ‘*qi*’ distention, heat stroke, vomiting, diarrhoea, indigestion, inhibited urination, urine opacity, swollen sores, bleeding, skin diseases, worm infection, blood dysentery, bone fracture, arthritis and paralysis.	Whole plant (fruits, bark, leaves, roots and root bark)	Decoction of pounded fresh/dry fruit taken orally to treat cough, diarrhoea, stomachache, toothache, bleeding. Paste of grounded root bark and leaves applied to relieve athlete’s foot pain and other skin diseases. Fresh ripe/unripe fruits consumed as tranquilizer and to relieve cold, cough, respiratory diseases, stomach problems and headache. Fruits oil aids recovery from paralysis. Leaves used to treat cholera. Aqueous mixture of pounded fruits and leaves, taken twice daily to treat blood dysentery, stomach problem and fever. Leaf paste applied on forehead as remedy for headache. Paste of bark applied to heal bone fracture. Seeds chewed to treat thread worm infections.	**Alkaloids**: (−)-8-*O*-Methyloblongine; (−)-Litcubine; (−)-Litcubinine; (−)-Magnocurarine; (−)-Oblongine; (+)-8-methoxyisolaurenine-*N*-oxide; (+)-Isoboldine β-*N*-oxide; (+)-*N*-(Methoxycarbonyl)-*N*-norboldine; (+)-*N*-(Methoxycarbonyl)-*N*-norglaucine; (+)-*N*-(Methoxycarbonyl)-*N*-norlauroscholtzine; (+)-*N*-(methoxylcarbonyl)-*N*-norglaucine; (+)-*N*-(methoxylcarbonyl)-*N*-norbulbodione; (+)-*N*-(methoxylcarbonyl)-*N*-nordicentrin; (+)-*N*-(methoxylcarbonyl)-*N*-norisocorydione; (+)-*N*-(methoxylcarbonyl)-*N*-norpredicentrine; Isoboldine; Atheroline; Boldine; Glaziovine; Isocorydine; Isodomesticine; Laurolitsine; Litebamine; N-Methyllaurotetanine; N-Methyllindcarpine; Norisoboldine; Norisocorydine Xanthoplanine **Monoterpenes**: Camphene; 1,8-Cineole; Citronellal; Citronellol; *p*-Cymene; Geranial (Citral a); Geraniol; Limonene (Cinene); Linalool; Myrcene; Neral (Citral b); β-Phellandrene; α-Pinene; β-Pinene; α-a-Isopulegol; Sabinene; β-Terpenene; α-Terpineol; α-Terpinyl acetate; Litseacubebic acid **Sesquiterpenes**: β-Caryophyllene; trans-Nerolidol **Diterpenoids**: Cubelin **Flavonoids**: Salvigenin **Amides**: cis-*N*-Feruloyl-3-methoxytyramine; *N-*Feruloyl-3-methoxytyramine; 3-Methoxy-*N*-sinapoyltyramine; *N-*trans-3,4-methylenecinnamoyl-3-methoxytyramine; Cubebamine A; 1,2-Dihydro-6,8-dimethoxy-7-1-(3,5-dimethoxy-4-hydroxyphenyl)-*N*1,*N*2-bis-[2-(4-hydroxyphenyl)ethyl]-2,3-naphthalene dicarboxamide; *N-cis*-3,4-methylenedioxycinnamoyl-3-methoxytyramine **Lignans**: Eugenol; Syringaresinol; 9,9′-*O*-di-(*E*)-feruloyl-(+)-secoisolariciresinol; 9,9′-*O*-di-(*E*)-feruloyl-5,5′-(+)-dimethoxysecoisolariciresinol; Balanophonin B; (+)-Medioresinol **Steroids**: β-sitostenone; Daucosterol; β-Sitosterol; Capric acid; *cis*-Dec-4-enoic acid; *cis-*Dodec-4-enoic acid (Linderic acid); *cis*-Tetradec-4-enoic acid (Tsuzuic acid); Hexadecenoic acid; Lignoceric acid; Lauric acid; Linoleic acid; Myristic acid; Oleic acid; Palmitic acid; Ethyl palmitate; Stearic acid; Ethyl stearate; Litseacubebic acid; 2,6-Dimethyl-6-hydroxy-2*E*,4*E*-hepta-2,4-dienal; 6,7-Dihydroxy-3,7-dimethyl-oct-2-enoic acid **Other compounds**: 2,5-Dimethoxy-*p*-benzoquinone; 2,6-Dimethoxy-*p*-benzoquinone; Vanillic acid; (6*R*)-3,7-Dimethyl-7-hydroxy-2-octen-6-olide; Cubebanone; Threo-2,3-bis(4-hydroxy-3-methoxyphenyl)-3-ethoxypro-pan-1-ol; Erythro-2,3-bis(4-hydroxy-3-methoxyphenyl)-3-ethoxypropan-1-ol	[42,43,44,45,46,47,48]
*Litsea glaucescens* Kunth *Laurel*, *aguarel*, *laurelillo* (Mexican), *Ecapatli* (Aztec)	Treats pain, central-nervous-system-related disorders such as epilepsy, depression, anxiety and fright, infections, fever, rheumatism, vomiting, diarrhea, cramps, indigestion, dysmenorrhea, sterility and colic Aids postpartum recovery.	Leaves	Infusion of leaves, scrubbing site with alcoholic extracts of leaves, vapor inhalation of the boiled or burned leaves. Used in baths for postpartum recovery.	Terpenes and phenolic compounds	[49,50]
*Litsea glutinosa* (Lour.) C.B. Rob. *Bois d’oiseaux* (Mauritian), *narra alagi*/*narra mamidi/Maidalakdi Lenja*/*Maadho saak*/*Papal* (Indian), *Medasak* (Sanskrit); Sablot (Philippines); *Chan Gao Mu Jiang Zi* (Chinese)	Analgesic, aphrodisiac, antiseptic, antispasmodic, demulcent and emollient. Remedy for diarrhoea, dysentery, gastroenteritis, indigestion, rheumatism, arthritis, sprain, bruises, wounds, sore, boil, abscess inflammation, oedema, swelling, backache, rheumatic and gouty joints, bone fracture, traumatic injuries, nervous crisis, haemorrhoids, allergies, colds and asthma. Promotes longevity, semen generation.	Bark, bud, leaves and seeds	Mucilaginous bark or decoction from fresh bark beneficial for diarrhoea, dysentery and rheumatism. Fine paste of ground bark and water is applied warm as a plaster to relieve bruises, sprain, inflammation, wounds, backache, bone fractures and rheumatic and gouty joints. Tea made from powder of 10–15 g dry bark taken at bed time for 2–4 days or mixture of maize flour, *Ghee* and *Gur* fried until brown in decoction water used to treat severe backache. Bud used to treat wounds. Leaf poultice as an emollient and for treating haemorrhoids, gastrointestinal disorder, joint pain (rheumatism) and allergies. Seed oil used to treat rheumatism.	Alkaloids, anthraquinones, cardiac glycosides, flavonoids, glycosides, phenols, saponins, steroids, tannins, terpenoids, volatile compounds, amino acids and carbohydrates **Alkaloids**: Boldine	[43,45,51,52,53,54,55,56,57]
*Litsea guatemalensis* Mez. *Laurel* (Mexican), *laurelillo*, *laurel silvestre*, *arrayán* (Spanish)	Treats fevers, headache, arthritis, stomachache, diarrhoea, emesis (vomiting), chills, throat infection, infectious diseases of the digestive system, urinary tract infection, broken bones, gastrointestinal diseases, skin conditions, trauma, muscular pain, rheumatism, stings, cultural affiliated syndromes, renal diseases, colic, swellings and disease of the circulatory and nervous system.	Leaves	Boiled leaf infusion used as remedies for fevers, headache, stomachache, diarrhoea, emesis and chills. Infusion gargled to treat throat infections. Paste of crushed leaves applied to treat arthritis. Used in baths for relieving fevers, chills, urinary tract infections and broken bones. Used as healing tonic for general health.	**Monoterpenoids**: dl-carvone; **Monoterpenes**: 1,8-Cineole; Linalool; α-Terpineol; **Flavonoids**: Pinocembrin; **Isoflavones**: 5,7,3′,4′-Tetrahydroxy-isoflavone; **Coumarins**: Scopoletin	[45,50,58]
*Litsea monopetala* (Roxb.) Pers or *Litsea polyantha* Juss. Menda khal (Bengali); Ngop (Indian)	Stimulant, astringent, spasmolytic, antidiarrheal, analgesic, antiseptic, antidepressant, anti-infertility, cytotoxic, antifungal, insecticide, purgative and laxative. Relieves pains, bruises, contusions, arthritis, stomachache, diarrhoea, dysentery, diabetes, dislocation, bone fractures, gonorrhoea, skin diseases and boils.	Leaves, bark, trunk and roots	Leaves useful against arthritis and bone cracks. Fresh green leaves used to treat diarrhoea and dislocation. Bark used as nerve and bone tonic, stimulant, analgesic and antiseptic and for treating stomachache and arthritis. Bark, leaves and roots used to gonorrhoea, skin diseases, boil, etc. Aqueous bark extract used to treat diarrhoea and dysentery. Pulverised/macerated bark applied to relieve pain due to blows, bruises, strenuous work or fractures. Pulverised roots applied externally for pains, bruises, contusions and swellings. Seed fat used in ointments for relieving rheumatism.	Phenols, alkaloids, butanolides, amides, butenolactones, steroid fatty acids, lignans, monoterpenes and sesquiterpenes **Phenols**: Eugenol; Chalcone **Sesquiterpenes**: Caryophyllene oxide; Humulene oxide **Fatty acids**: Capric acid; Myristic acid	[45,59,60,61,62,63,64,65]
*Litsea coreana* var.* sinensis* (C.K. Allen) Yen C. Yang & P.H. Huang	Relieves stomachache and pain and treats traumatic injury.	Roots and leaves	Roots are used to relieve stomach pain, and the leaves are used to treat pain and traumatic injuries	**Monoterpenoids**: Menthane; **Sesquiterpenes**: Farnesane; Copaene; Aristolone; Cubebane; Cedrane; α-Patchoulene	[45]
*Litsea deccanensis* Gamble	Alleviates chest pain.	Leaves	-	**Alkaloids**: Boldine; Corytuberine; Dicentrine; Isocorydine; Laurolitsine; Magnoflorine; Nordicentrine.	[45]
*Litsea elliptica* Blume *Medang perawas* (Indonesian), *Pawas* (Bruneian), Tham-mung (Thailand)	Treats headaches, cancer, stomach ulcers and fever.	Leaves	Crushed leaves applied to forehead to treat headaches.	**Steroids**: Palmitic acid, methyl ester; Linoleic acid, methyl ester; **Diterpenoids**: Phytol; **Other compounds**: Catechol; Mono(2-ethylhexyl) phthalate; *dl*-α-Tocopherol	[66,67,68]
*Litsea euosma* W.W. Smith or *Litsea mollis* Hemsl. (TBC) *Fourflower Litse*, *Qing Xiang Mu Jiang Zi* (Chinese).	Carminative, diuretic, expectorant, stimulant, stomachic, antiasthmatic, arthritis, sedative, antidysenteric and antiseptic. Treats stomachache, abdominal distention, dyspepsia, spleen dropsy/oedema, arthritis, emesis (vomiting) and diarrhoea. Dispels wind and moves qi, fortifies spleen and disinhibits damp and resolves toxins.	Fruits, roots and leaves	-	**Alkaloids**: Laurolitsine; **Monoterpenes**: 1,8-Cineole; Geranial (Citral a); Neral (Citral b); Limonene; Citronellal; Linalool; α-Pinene; β-Pinene; (*E*)-β-Ocimene; (*Z*)-β-Ocimene; Germacrene; **Sesquiterpenes**: Farnesane; Oploanane; Bourbonane; Cedrane; **Flavonoids**: Astragalin (Kaempferol3-*O*-β-d-glucopyranoside); Dihydrodehydrodiconiferyl alcohol; **Steroids**: Stigmasterol; 6-*O*-Palmitoyl-β-sitosteryl-d-glucoside; **Fatty Acids**: Docosanoic acid; Hexacosanoic acid; Triacontanoic acid; **Other compounds**: Euosmoside A; Euosmoside B; 5-Hydroxy-6-methyl-3-(undec-10-enyl)-5,6-dihydropyran-2-one	[43,45]
*Litsea garciae* S. Vidal	Antifungal and antioxidant. Treats caterpillar stings, boils, rectal bleeding, skin infections, diseases and burns, beri-beri, sprains, muscular aches and snake bites.	Leaves and bark	Ground bark used as dressing for treating caterpillar stings and boils. Decoction of bark taken to treat rectal bleeding. Poultice of leaves and young shoots mixed with shallot and fennel seeds applied to treat skin infections, diseases and burns. Warm poultice of leaves applied to treat beri-beri. Poultice of root bark applied to cure the sprains. Pounded and warmed bark used to treat muscular aches and sprains. Combination of *L. garciae* and durian bark used as antidote for snake bites.	**Alkaloids**: Actinodaphnine; Boldine; Isodomesticine; Laurolitsine; Reticuline; **Monoterpenes**: 1,8-Cineole; Geraniol; **Sesquiterpenes**: γ-Cadinene	[45,69]
*Litsea. garrettii Gamble*	Heat-clearing, detoxifying, detumescence and analgesic. Relieves jaundice and itching, eliminates parasites, wind and dampness.	Roots	-	-	[45]
*Litsea lancilimaba*	Relieves chest pain, chest tightness, asthma, coronary heart disease and angina pectoris.	-	-	**Monoterpenes**: Cineole **Sesquiterpenes**: Copaene; Ylangene; Cubebane; Cedrane; Farnesane	[35,45]
*Litsea moupinensis* var. *szechuanica* (C. K. Allen) Yen C. Yang et P.H. Huang	Carminative, diuretic, expectorant, stimulant, stomachic, antiasthmatic, antiarthritis, sedative, antidysenteric and antiseptic.	Fruit	-	-	[45]
*Litsea pedunculata* (Diels) Y.C. Yang & P.H. Huang	Relieves gastroenteralgia, edema and rheumatic arthritis.	Stem bark	-	**Triterpenoids**: Betulin; **Flavonoids**: Alpinetin; Flavokawin B; Linderol A; Litseaone B; Pinocembrin; Quercetin	[45]
*Litsea populifolia* (Hemsl.) Gamble	Treats stomachache, dyspepsia, relieves pain, indigestion, nausea and emesis (vomiting).	Fruits and leaves	-	**Monoterpenes**: Citral; Limonene; Nerol; 1,8-Cineole; α-Pinene; β-Pinene; Linalool; **Sesquiterpenes**: Caryophyllene; **Terpenoids**: Camphor; **Other compounds**: Methylheptenone	[45]
*Litsea pungens* Hemsl. *Zhen Cai* (Chinese)	Strengthens spleen. Relieves dyspepsia, diarrhea, sunstroke, sore, scab, stomach distension, pain, stomachache, arthralgia, influenza, cough of phlegm-rheum and beri-beri.	Fruits, leaves, stems and roots	-	**Alkaloids**: Launobine; Laurotetanine **Monoterpenes**: 1,8-Cineole; (*R*)-Limonene; Neryl acetate; **Sesquiterpenes**: Aromadendrene; **Monoterpenoids**: Carvone; **Flavonoids**: Pinocembrin; Pinostrobin; 2-(Hexahydro-1,3-benzodioxol-5-yl)-3,4-dihydro-5,7dimethoxy-2H-chromen- 3-ol; 2’,6’-Dihydroxy-4’-methoxychalcone; **Steroids**: Daucosterol; **Fatty acids**: *cis*-4-Decenoic acid, *cis*-4-Dodecenoic acid; *cis*-4-Tetradecenoic acid; **Other compounds**: 5,6-Dehydrokawain; Palmitone	[43,45]
*Litsea rotundifolia* Hemsl.	Treats rheumatic pain.	Roots	-	**Alkaloids**: Boldine; Laurolitsine; *N-*Acetyllaurolitsine	[45]
*Litsea rotundifolia* var.* oblongifolia* (Nees) C.K. Allen	Treats edema, rheumatic arthritis and stomach disorder.	Fruits, leaves, roots and bark	-	**Alkaloids**: Boldine; Laurolitsine; Butenolactones: Lincomolide A; Lincomolide C; Litsenolide A1; Marliolide; Rotundifolide A; Rotundifolide B; **Fatty acids**: Undecanoic acid; Lauric acid; Myristic acid; Palmitic acid; 13-Tetradecenoic acid; 11-Dodecynoic acid; 13-Tetradecynoic acid; **Other compounds**: Oblongifolinol; Rotundifolinol	[45]
*Litsea rubescens* Lecomte	Relieves gastroenteralgia, enterogastritis, edema, rheumatic arthritis, stomachache and dyspepsia.	Stem, bark, roots and fruits	-	**Flavonoids**: Alpinetin; Flavokawin B; Linderol A; Litseaone A; Pinocembrin; Quercetin	[45]
*Litsea sebifera* Pers.	Treats urinary problems and rheumatic arthritis.	Stem, root and leaves	Extract of stem or root consumes 2–3 times a day to treat urinary problems. Paste made from leaves applied to affected area to treat rheumatic arthritis.	**Alkaloids**: Boldine; Laurotetanine; Litseglutine A (Litseferine); *N*-Methyllaurotetanine; Sebiferine	[45,70]
*Litsea veitchiana* Gamble	Treats indigestion, gastroenteralgia and dyspepsia.	Fruits	-		[45]
*L. verticillata* Hance	Treats rheumatism, dissipates stasis and relieves menstrual cramps, pain, painful swellings from knocks and falls, stomachache, wind-damp impediment pain, soreness, fractured bones and snake bites.	Roots and leaves	-	**Butanolides and Butenolactones**: 4-Hydroxy-2-methylbut-2-enolide; Hydroxydihydrobovolide; Litseabutenolide; **Sesquiterpenes**: Aphanamol II; 10-Hydroxy-15-oxo-a-cadinol; Chromolaevanedione; Eudesm-4(15)-ene-1β,6α-diol; 5-epi-Eudesm-4(15)-ene-1β,6β-diol; 7-epi-Eudesm-4(15)-ene-1α,6α-diol; 7-epi-Eudesm-4(15)-ene-1β,6β-diol; Isolitseane A; Isolitseane B; Isolitseane C; Litseachromolaevane A; Litseachromolaevane B; Litseagermacrane; Litseahumulane A; Litseahumulane B; Litseaverticillol A; Litseaverticillol B; Litseaverticillol C; Litseaverticillol D; Litseaverticillol E; Litseaverticillol F; Litseaverticillol G; Litseaverticillol H; 1,2,3,4-Tetrahydro-2,5-dimethyl-8-(1-methylethyl)-naphthalene-1,2-diol; Octahydro-4-hydroxy-3a-methyl-a-(1-methylethyl-7-methylidene-1*H*-in- dene-1-methanol; Oxyphyllenodiol B; Verticillatol; **Lignans**: (+)-Epiexcelsin; (+)-5′-Demethoxyepiexcelsin	[43,45,71]
*Litsea zeylanica* Nees and T.Nees	Dispels wind and relieves pain and rheumatism.	Roots	-	**Alkaloids**: Norisoboldine; Reticuline; **Monoterpenes**: Linalool; α-Pinene; Terpinen-4-ol; **Sesquiterpene**: β-Caryophyllene	[45]

## 2. Methods

To identify and assess scientific evidences on the analgesic effects of plants from the *Litsea* genus, an extensive literature search using a systematic approach was conducted across multiple electronic databases including PubMed, Science Direct, Scopus and SpringerLink. Articles from the inception of the database to 7 February 2022 were gathered and evaluated. The search terms and strategies used in this study included “litsea” AND “analgesic” OR “antinociceptive” OR “pain relief”. We limited our search focus to only include original research papers that have conducted primary studies on the analgesic effects of plants from the *Litsea* genus. Articles eligible for inclusion in this systematic review were selected based on the following inclusion and exclusion criteria. 

The inclusion criteria are as follows:

1. Articles in the form of research articles, conference proceedings, technical papers or monographs;
2. Studies published in the English language only;
3. Studies that have demonstrated relevant biological activities pertaining to analgesia, antinociception or pain relief in any *Litsea* species;
4. In vitro laboratory studies, in vivo animal model studies or clinical trials on human candidates.

The exclusion criteria are as follows:

5. Studies that reported other pharmacological properties of the *Litsea* species irrelevant to pain alleviation;
6. Non-full text articles with minimum information on methodology and results;
7. Non-English language articles;
8. Reviews, letters, case studies, opinions, reports or editorial papers.

**Figure 1:**
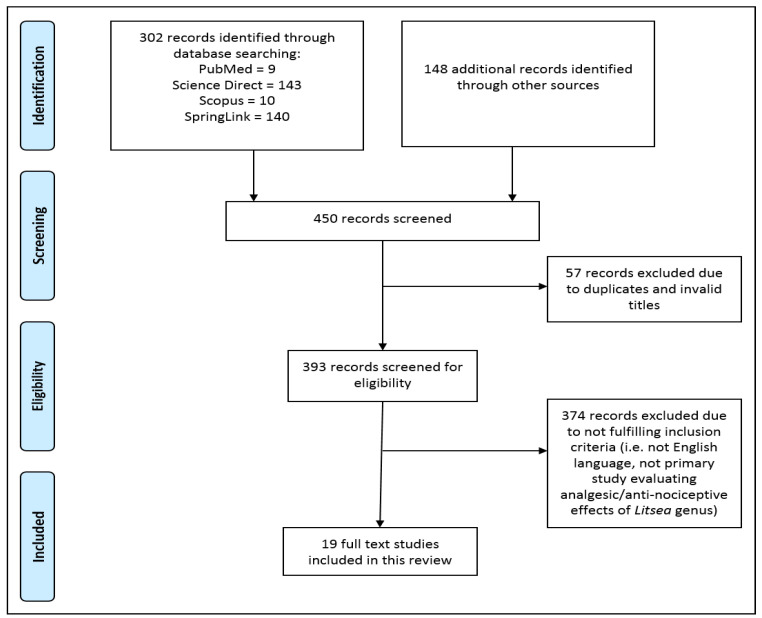
PRISMA flow diagram for selection of study for the systematic review.

The PRISMA flow diagram shown in Figure 1 was used as a guide to screen and select articles from the database.

## 3. Results

Based on our search strategy as shown in Figure 1, a total of 450 records were identified from the literature search where 57 were excluded due to duplications and invalid titles which mainly included indices of books and encyclopedias. Further screening was conducted to exclude another 374 studies that did not meet the set inclusion criteria. The remaining 19 studies were retrieved and included in this review, revealing a total of nine *Litsea* species that demonstrated some form of analgesia or associated effects via various in vivo, in vitro and clinical studies. Table 2 summarizes the findings from the studies according to the plant species and the extracts used.

### 3.1. Litsea cubeba (Lour.) Pers.

*Litsea cubeba* (Lour.) Pers. is an aromatic herb that is widely distributed in Southern China, Japan, Southern Asia and Southeast Asia [42,46,83]. The plant is valued for the sweet-scented, citral-rich essential oil produced from its fruits, which has economic importance in the pharmacy, cosmetics, perfumery, cookery and insecticide industries [83,84]. The fresh fruits are edible and are often used as a spice, while the fruits and seeds are commonly used as condiments [48].

Based on traditional Chinese medicine (TCM), *L. cubeba* has been used as a herbal medicine for warming the body’s core, improving blood circulation and relieving pain, asthma, chronic bronchitis, food accumulation, ‘*qi*’ distention, vomiting, diarrhea, abdominal pain, inhibited urine, urine opacity, swollen sores and toothache [43]. More specifically, Hong et al. (2015) [44] described that the decoction of the pounded fresh or dry fruit can be consumed to relieve cough, diarrhea, stomachache, toothache and bleeding. In India, fresh ripe or unripe fruits are consumed as a remedy for headache, cold, cough, respiratory diseases, stomach problems and sleep problems [46,48]. In addition, oil from the fruits was believed to be beneficial for paralysis, whereas the leaves were claimed to treat cholera [46]. According to Srivastava (2009) [48], people from the Adi tribe of Arunachal Pradesh consumed an aqueous mixture of the pounded fruits and leaves twice daily to treat blood dysentery, stomach problems and fever and chewed on the seeds to treat threadworm infections. Furthermore, a paste of the leaves and bark was applied to treat headaches and bone fractures, respectively [48]. Based on Nepalese traditional folklore, paste made from the ground leaves and root bark can be applied to relieve athlete’s foot pain and other skin diseases [47]. In general, the entire plant, including the fruits, bark, leaves and roots, has been suggested to possess analgesic, anti-asthmatic, anti-dysenteric, anti-inflammatory, antiseptic, astringent, carminative, diuretic, expectorant, hair-tonic, hypotensive, insecticidal, sedative and stomachic properties and it has been used to treat worm infection, arthritis, blood dysentery, cold, cough, indigestion, headache and bone fracture in traditional medicine [42,45]. 

More than 90 compounds have been reported for *L. cubeba* including various alkaloids, monoterpenes, sesquiterpenes, diterpenoids, flavonoids, amides, lignans, steroids and other compounds [45]. Of these compounds, geranial (Citral A), neral (Citral B), β-phellandrene, d-limonene, cubebanone, sabinene, citronellal, *cis*-4-Decenoic acid, isocorydine, laurolitsine, magnocurarine, β-terpenene and myrcene were frequently characterized in *L. cubeba* [42,43]. The oil of *L. cubeba* reportedly contains up to 70% of citral and has thus garnered a lot of interest as a commercially vital crop source for the production of citral, which is otherwise widely extracted from *Cymbopogon citrates* (lemongrass) [85]. Citral is a monoterpene that has been used as an antispasmodic, analgesic, anti-inflammatory, antipyretic, diuretic and sedative in traditional medicine [85,86]. It was confirmed to reduce neuronal excitability via inhibitory neurotransmitter modification, which, in turn, aids anxiety, insomnia, convulsion, pain and cognitive deficits [86]. In addition to citral, aporphine alkaloids and lignans are also considered major active components identified in the fruits and roots of *L. cubeba* due to their anti-inflammatory, antithrombotic, analgesic and antinociceptive properties [87,88]. Overall, the reported bioactivities of the major constituents in addition to the ethnomedicinal uses of *L. cubeba* suggest that the plant possesses substantial anti-inflammatory and neuropharmalogical potential especially for alleviating pain and other related ailments. 

The neuropharmalogical activities of the essential oil from the *L. cubeba* fruit have been investigated by Chen et al. (2012) [72]. Results from the tail-flick test of pain response showed that the 500 mg/kg by weight (b.w.) dose displayed maximum effect at 60 min post-treatment, which was comparable to the positive control, acetaminophen (90 mg/kg b.w.). Meanwhile, 100 and 300 mg/kg b.w. of the essential oil did not significantly prolong the reaction time in the treated mice. The researchers concluded that a high dose of the *L. cubeba* fruit oil was necessary to induce remarkable antinociceptive activity and suggested that the analgesic effect was due to the presence of neral, geranial and limonene, which were identified as the major constituents of the fruit essential oil [72]. In addition to the enhanced pain resistance, Chen and his co-workers (2012) [72] also showed that the essential oil could increase the pentobarbital-induced sleeping time and reduce stress in the treated mice based on the elevated plus maze test for anxiety. 

The analgesic property of the *L. cubeba* fruit oil was also investigated via the acetic-acid-induced writhing and hot plate test methods by a separate group [21]. In the acetic-acid-induced writhing test, dose-dependent decreases in the number of torsions were observed in the mice treated with 50, 100 and 200 mg/kg b.w. of the essential oil. However, all three doses of the essential oil did not increase pain resistance in the treated mice following heat stimulation, implying that the analgesic activity of the *L. cubeba* fruit essential oil was mainly via the peripheral mechanism of pain inhibition [21]. As acetic-acid-induced analgesia is associated with the release and augmented peritoneal fluid levels of inflammatory mediators such as histamine, serotonin, cytokines and eicosanoids [89], the antinociceptive action of *L. cubeba* fruit oil is likely due to its ability to reduce the inflammatory mediators or block the receptors involved. Indeed, the study showed that the fruit oil of *L. cubeba* was able to reduce serum levels of the proinflammatory cytokines tumor necrosis factor (TNF)-α, interleukin (IL)-1β, -6, -8 and -17A, while increasing the level of the anti-inflammatory cytokine, IL-10 [21]. This revealed that the *L. cubeba* fruit oil was able to induce peripheral analgesia via the anti-inflammatory response.

As mentioned earlier, Chen and his colleagues (2012) showed that a high dose of 500 mg/kg b.w. was required for the essential oil to induce observable antinociceptive activity in the tail-flick test. Both the tail-flick and hot plate approaches are model test methods for detecting central analgesic activity [89]. Both tests measure latencies in nociceptive response to thermal stimulus whereby the tail flick is mainly a spinal response, while the hot plate test measures the supraspinal antinociceptive response [90]. Considering that pain perception was not reduced at the maximum dose of 200 mg/kg b.w. of the fruit oil in the hot plate test by Zhao et al. (2020) [21] and that only a high dose of 500 mg/kg b.w. was able to induce analgesia in the tail-flick test [72], it suggests that a concentration threshold of the *L. cubeba* fruit oil must be reached in order to activate central analgesia at the spinal and supraspinal level. 

### 3.2. Litsea elliptibacea Merr.

*Litsea elliptibacea* Merr. is a plant that is endemic to the tropical forest of Sabah, Malaysia [91,92]. The ethnobotanical properties and uses of the plant have not been previously described. 

Thus far, only two records have been identified that investigated the pharmacological potential of the plant. One of the studies reported the in vitro anti-plasmodial potential of the *L. elliptibacea* crude leaf extract against the chloroquine-resistant strain (Gombak A) of *Plasmodium falciparum*, indicating its potential as a candidate for development into an alternative therapy to anti-malarial drugs [91]. 

Another study by Chung et al. (2005) [73] investigated the neurobiological activity of *L. elliptibacea* amongst 184 other plants sampled from the Malaysian forest. The results from the study showed that the bark extract of *L. elliptibacea* caused 84 ± 1% inhibition of specific binding of the radioligand, [^3^H]-6-OH-DPAT, to the 5-hydroxytryptamine 1A receptor (5-HT_1A_R) [73,93]. 

Serotonin or 5-hydroxytryptamine receptors (5-HTRs), which are widely expressed throughout the CNS, have been commended as innovative targets for analgesic drugs as they have complex involvements in pain modulation [94,95]. Specifically, full and partial binding to 5TH_1A_R has been shown to induce effective pain relief by inhibiting the transmission of nociceptive signals [94,95,96,97,98,99,100]. Considering the high receptor-binding activity of the *L. elliptibacea* bark extract to 5HT_1A_R, it suggests that the bark extract could in turn cause analgesia and instigate pain relief. 

Six alkaloids have been isolated from the ethanolic extract of the *L. elliptibacea* stem bark including (+)-*N*-methylactinodaphnine, (+)-actinodaphine, (+)-*N*-methyllaurotetanine, (+)-boldine, (+)-norboldine and (+)-reticuline [101]. In addition to alkaloids, phytochemical analyses have also demonstrated the presence of saponins in the fresh leaf and bark extracts of *L. elliptibacea* [91]. Nevertheless, the bioactive constituents responsible for the reported central nervous system (CNS) activity has yet to be identified [93]. 

### 3.3. Litsea japonica (Thunb.) Jussieu 

*Litsea japonica* (Thunb.) Jussieu is an evergreen broadleaf tree species that thrives in the Southern province and islands of Korea and South Asian regions including Japan [102]. Reports on the traditional use of the plant in folk medicine are limited. However, various biological studies have shown that the extracts from various parts of *L. japonica* possess analgesic [24], anti-inflammatory [103], anti-complement [104] and antidiabetic properties [105] and are prospective therapies for diabetic retinopathy [106] and osteoarthritis [74].

According to a recent review by Azhar and Salleh (2020) [107], 20 and 25 compounds have been reported in the essential oils of the seed and mesocarp of the *L. japonica* fruit respectively, where Germacrene D and caryophyllene were identified as the major constituents. Various lactones including akolactones A, B, D and E, hamabiwalactones A and B, lincomolide C, lisealactones H_1_ and H_2_, litseakolide B, litsealactones A and B and litsenolides A_1_, A_2_, B_1_, B_2_, C_1_, C_2_, D_1_, D_2_, E_1_, E_2_ and F_1_ were also isolated from various parts of the plant [103,108,109,110]. In addition, four flavonoids including epicatechin, afzelin, quercitrin and tiliroside, were isolated from *L. japonica* leaves [104]. Potent anti-complement activities were reported for hamabiwalactone B, akolactone B, afzelin, quercitrin and tiliroside with IC_50_ values of 149, 58, 258, 440 and 101 μM, respectively [104,108]. Meanwhile, hamabiwa A and B lactones have exhibited significant antinociceptive activity in both peripheral and central nervous models of pain [24]. In another study, litsenolides A_1_, A_2_, B_1_, B_2_, D_2_, E_2_ and F_1_, litseakolide B and lincomolide C demonstrated potent nitric oxide (NO) inhibition in lipopolysaccharide (LPS)-stimulated RAW264.7 cells [103]. Furthermore, litseakolide B and litsenolides A_2_, B_2_ and F_1_ downregulated inducible nitric oxide synthase (iNOS) and COX-2 expression while litsenolide A_2_ suppressed the mRNA expression of iNOS, COX-2, IL-6 and TNF-α [103]. The reported anti-complementary, anti-inflammatory and antinociceptive effects of the bioactive components of *L. japonica* signify that the plant may have therapeutic benefits against hyperalgesic, inflammatory and degenerative diseases.

The analgesic potential of *L. japonica* fruits was demonstrated by the antinociceptive activities of its 30% ethanolic extract, CH_2_Cl_2_ fraction and active constituents (hamabiwalactones A and B) in both in vivo peripheral and central nervous pain models [24]. In the acetic-acid-induced writhing test for peripheral analgesia in mice, pre-treatment with 50 and 100 mg/kg b.w. of the ethanolic extract significantly reduced the writhing frequency by 60.8% and 78.5%, respectively. Similar dose-dependent writhing inhibition was also observed in the CH_2_Cl_2_ fraction-treated (82.6% and 88.4%), hamabiwa A-treated (67.5% and 81.0%) and hamabiwa B-treated (64.3% and 72.3%) groups. In addition to peripheral nociception, the extract, fraction and active components of *L. japonica* fruit also suppressed the central nervous pain response as they significantly increased the latencies and basal pain thresholds in both the tail-flick and hot plate tests. In all three models of pain, the CH_2_Cl_2_ fraction demonstrated maximal efficacy while exhibiting minimal cytotoxicity. Considering the association between the nociceptive response and inflammatory pain, the anti-inflammatory effects of the extract and fraction were further investigated. Both suppressed the release of inflammatory mediators, COX-2/prostaglandin E2 (PGE_2_) and NO/iNOS, and pro-inflammatory cytokines (IL-1, IL-6 and TNF-α) in LPS-stimulated RAW264.7 cells. Based on these findings, Koo and colleagues (2014) [24] proposed that the suppressed c-Jun N-terminal kinase (JNK)/p38 mitogen-activated protein kinase (MAPK) and inhibited nuclear factor-kappa B (NF-κB) signaling pathway may be involved in the anti-inflammatory and anti-nociceptive actions of the *L. japonica* fruit extract and fraction, induced by the lactones hamabiwa A and hamabiwa B.

The pain-relieving effect of the ethanolic extract of the *L. japonica* fruit was also demonstrated in the randomized, double-blinded, placebo-controlled study conducted by Ahn et al. (2017) [74] to evaluate the efficacy of the extract against knee osteoarthritis. The study showed that a 12-week-long consumption of the fruit extract ameliorated the scores for pain, stiffness and knee function along with decreased blood matrix metalloproteinase-9 (MMP-9) levels in subjects with painful knee osteoarthritis. MMPs, which include collagenases (MMP-1, MMP-8 and MMP-13), gelatinases (MMP-2 and MMP-9) and stromelysin-1 (MMP-3), are zinc-dependent endopeptidases that can degrade native collagens and proteoglycans [111]. The elevated expression and activity of the proteinases have been implicated in the manifestation of osteoarthritis as they can cause the inappropriate degradation of articular cartilage as a result of stress-induced intracellular signals, catabolic cytokines and inflammatory mediators released by synovial cells and chondrocytes [112,113]. Although blood MMP-3, TIMP-1, COMP, IL-6 and fructosamine levels and urinary CTX-II levels remained unchanged in the *L. japonica* fruit extract-treated groups, the marked dose-dependent reduction in plasma levels of MMP-9 suggested that the improvements in pain, stiffness and joint function observed in the treatment groups were due to reduced cartilage destruction via MMP-9 inhibition by the *L. japonica* fruit extract [74].

In a separate study on the anti-osteoarthritic effect of n-hexane extract of *L. japonica* fruit using monosodium iodoacetate (MIA)-induced osteoarthritic rat models, decreased expression of MMP-2 and MMP-9 in the joints and infiltration of inflammatory cells into the synovium were reported [114]. In addition, the extract also inhibited proinflammatory cytokines including TNF-α, IL-1 and IL-6 in the joints and serum of the treated rats, as well as NO, PGE_2_, IL-6 and TNF-α production in LPS-activated macrophages. Similar to the findings of Koo and colleagues (2014) [24], Kim et al. (2017) [114] also suggested that the *L. japonica* fruit extract was able to suppress the phosphorylation of p-38 MAPK and JNK as a means to relieve the painful symptoms of osteoarthritis. Overall, the results from the studies emphasized that the analgesic pharmacology of the *L. japonica* fruits is closely associated with the anti-inflammatory actions of the plant.

### 3.4. Litsea glaucescens Kunth 

*Litsea glaucescens* Kunth is a small tree species native to Central America and Mexico where it is commonly referred to as ‘*laurel*’, ‘*aguarel*’, ‘*laurelillo*’ or ‘*ecapatli*’ [45,49]. In Mexico, the leaves of the plant are highly sought after for its culinary use as a condiment and a folk remedy for various central nervous system-related disorders such as epilepsy, depression, anxiety and fright, as well as pains, infections, fever, rheumatism, emesis, diarrhea, cramps, indigestion, dysmenorrhea, postnatal pain, infertility and colic [45,49,50]. The plant has also been used to facilitate child birth. Traditionally, the remedies were taken in the form of leaf infusions, baths or steam baths [50]. Otherwise, it could also be inhaled as vapor from the boiled or burned leaves. Alcoholic extracts of the leaves may also be applied or rubbed on the affected sites to relieve the painful symptoms [50].

López-Romero et al. (2018) [49] attributed the ethnomedicinal activities of *L. glaucescens* to the phenolic and terpene content of the plant, whereas Jiménez-Pérez et al. (2009) [50] related the folkloric use of *L. glaucescens* to its high composition of 1,8-cineole (eucalyptol). Indeed, phenolic compounds including epicatechin, quercitrin and kaempferol, have been isolated from the methanolic extract of the leaves and were identified as the major constituents of the most polar fraction of the extract [115]. Moreover, the flavonoids pinostrobin and pinocembrin, as well as 2′,6′-dihydroxy-4′-methoxydihydrochalcone, were also isolated from the ethanolic extract of the bark of *L. glaucescens* [115]. Meanwhile, monoterpenes including eucalyptol (26.06%), *o*-cymene (25.86%), α-pinene (3.86%), linalool (3.64%), γ-terpinene (2.83%), β-pinene (2.34%) and carvone (1.85%) were also characterized in the essential oil of the *L. glaucescens* leaves amongst the 45 compounds identified [116]. Although these monoterpenes in addition to limonene (8.66%), terpinen-4-ol (5.08%), neryl acetate (1.35%) and carveol (1.35%) made up a major portion (82.88%), only linalool and β-pinene showed potent antidepressant activity similar to the positive control, imipramine (30 mg/kg b.w.) [116]. The presence of the aforementioned phenolic compounds has been associated with the high antioxidant activity observed in the methanolic leaf extract of *L. glaucescens* [115], whereas 1,8-cineole has reported anti-inflammatory and antinociceptive effects [117]. The presence of these bioactive compounds thus infers the potential pharmacological benefits of *L. glaucescens* against analgesia and also neurogenic, inflammatory and oxidative-stress-derived ailments.

Although the analgesic effects of *L. glaucescens* have yet to be elucidated through pharmacological studies, antinociceptive studies on 1,8-cineole [117], a major constituent of *L. glaucescens* [50,116], have revealed potential analgesic activity for the plant. According to the study by Santos and Rao (2000) [117], an oral dose of 100–400 mg/kg b.w. of 1,8-cineole could inhibit nociception induced by the intraplantar and intraperitoneal administration of formalin and acetic acid, suggesting the involvement of peripheral analgesic mechanisms. A non-opioid system was implicated in the analgesic activity as pretreatment with naloxone (a µ-opioid receptor antagonist) did not reverse the antinociceptive effect of 1,8-cineole [117]. This suggests that the analgesic effect was due to the anti-inflammatory action of the compound against inflammation-stimulated analgesia [89]. Indeed, 1,8-cineole has been shown to attenuate inflammation in LPS-stimulated monocytes via inhibition of the pro-inflammatory mediator and cytokines, including leukotriene B4 (LTB4), PGE_2_, TNF-α, IL-1β and thromboxane B2 (TxB_2_) [118]. Thus, this suggests that *L. glaucescens* may have the potential to induce antinociceptive activity via the non-opioid anti-inflammatory pathway due to its high composition of 1,8-cineole.

In a separate study, Guzmán-Gutiérrez and co-workers (2012) [116] demonstrated the antidepressant activity of the *L. glaucescens* leaf essential oil and its active principles, linalool and β-pinene, in mice using the forced swimming test (FST). Since the essential oil did not alter the spontaneous locomotor activity of the mice as shown in the open field test (OFT), it indicated that the reduced immobility time of the treated mice in the FST was not due to increased motor activity and that a genuine antidepressant effect was instigated by the *L. glaucescens* essential oil [116]. In contrast, decreased spontaneous locomotor activities were observed following the administration of linalool and β-pinene, possibly due to sedative effects of the monoterpenes as the exploratory activity of mice was also reduced following linalool and β-pinene treatment in the exploratory cylinder test (ECT). Subsequent work by Guzmán-Gutiérrez et al. (2015) [75] revealed that the antidepressant action of linalool and β-pinene occurred via the serotonergic or 5-HT pathway via interaction with postsynaptic 5-HT_1A_R. In addition to antidepressant effects, the activation of the serotonergic pathway has also been implicated in the modulation of nociception as mentioned earlier (refer to the section on *L. elliptibacea*). As agonists of 5-HT_1A_R have been reported to inhibit nociceptive signal transmissions to instigate pain relief [94,96,97,98,99,100], it suggests that the analgesic effects of *L. glaucescens* in traditional medicine may also be due to the interaction of linalool and β-pinene with 5-HT_1A_R.

### 3.5. Litsea glutinosa (Lour.) C.B. Rob.

*Litsea glutinosa* (Lour.) C.B. Robinson is a medium to large-sized semi-evergreen aromatic tree species that can grow up to 20 m in height [51,52,53]. It is vastly distributed in various subtropical and tropical habitats worldwide including India, South China, Bhutan, Nepal, Thailand, Myanmar, Vietnam, Malaysia, the Philippines, Australia and the Western Pacific Islands [51,53]. Despite its wide distribution, it has been listed as a critically endangered species in India and the Philippines as a result of over-exploitation due the popular use of its bark in indigenous medicine to treat various ailments [53,119,120]. 

Various parts of the plant, including the bark, bud, leaves and seed, have been used as an analgesic, aphrodisiac, antiseptic, antispasmodic, demulcent and emollient as well as a remedy to treat diarrhea, dysentery, gastroenteritis, indigestion, rheumatism, arthritis, sprain, bruises, wounds, sores, boils, abscess, inflammation, oedema, swelling, backache, rheumatic and gouty joints, bone fracture, traumatic injuries, nervous crisis, hemorrhoids, allergies, colds and asthma [43,51,52,53,54,56]. Specifically, mucilaginous bark or a decoction from the fresh bark or stem bark are taken to relieve diarrhea, dysentery and rheumatism [51,52,57]. The bark can also be ground and mixed with warm water to form a fine paste for application as a plaster to relieve pain, bruises, sprain, inflammation, wounds, backache, bone fractures and rheumatic and gouty joints [51,52,55]. For the treatment of severe backache, a mixture of maize flour and fried *Ghee* and *Gur* in the decoction water of the bark or tea made from the powder of 10–15 g dry bark was taken at bed time for 2–4 days [56]. The bud of *L. glutinosa* has also been used as a remedy for wounds, whereas the seed oil has been used to treat rheumatism [45,54]. A poultice of the leaves has also been used as an emollient and a remedy for hemorrhoids, gastrointestinal disorder, joint pain (rheumatism) and allergies [54]. According to some reports, a decoction of the bark sap is taken to promote longevity [51,52].

Multiple studies have unveiled the presence of abundant phytochemicals in various extracts of the leaves, bark and fruit, leaf and seed oils of *L. glutinosa* as summarized in the review by Chawra et al. (2021) [53]. Some of the most commonly identified constituents in *L. glutinosa* include alkaloids, flavonoids, saponins, tannins, terpenoids and glycosides [3,25,33,43,52,121,122,123,124,125,126]. *N*-methylactinodaphnine, boldine, *N*-methyllaurotetanine and isoboldine are four aporphine alkaloids isolated from the plant by activity-guided fractionation [121]. Meanwhile, a benzoic acid derivative, eusmoside C, and four butenolides, (3*R*,4*S*,5*S*)-2-hexadecyl-3-hydroxy-4-methylbutanolide, litsealactone C, litsealactone D and litsealactone G, were isolated and characterized from the methanolic extract obtained from the heartwood of *L. glutinosa* [127]. Two phytosterols, stigmasterol and α-sitosterol, were also isolated from the ethanolic extract of the bark [115]. According to Parikh and Rangrez (2012) [123], alkaloids represented the majority of the phytochemicals characterized in the methanolic extract of *L. glutinosa* bark. The team also highlighted the presence of oleic acid, eicosanoids and phytoestrogens-like pregene derivative and androsta-triones which have been recognized for their hypotensive, anti-inflammatory and osteoprotective potentials. A separate investigation further demonstrated that the essential oil obtained from the stem bark mainly constituted 9,12-octadecadienoic acid (62.57%), hexadecenoic acid (12.68%), stigmast-5-en-3-ol (6.87%) and vitamin E (2.51%) [128]. The presence of these characterized compounds suggests extensive pharmacological potentials for the plant including, but not limited to, antinociceptive, anti-inflammatory, anticancer, antioxidant, anthelminthic, antimutagenic, anti-ageing, antidiabetic, anti-dermatitic, anti-leukemic, hepatoprotective, hypocholesterolemic, anti-ulcerogenic, vasodilator, antispasmodic, anti-bronchitic and anticoronary activities [129,130,131,132,133,134]. This corroborates the substantial folkloric use of the plant to heal ailments, especially pain, inflammation and infections, as described earlier. 

Investigations on pain inhibitory potentials have been conducted on various extracts of *L. glutinosa* bark and leaves mainly using the hot plate and acetic-acid-induced writhing test models for central and peripheral nociception. Based on the hot plate test for central analgesia, the ethanolic extract of the bark was able to improve the pain threshold of the treated mice as early as 15 min following treatment with 100 and 300 mg/kg b.w. of the extract [79]. As for the leaves, two studies reported significant analgesic activities whereby a dose of 100 mg/L of the methanolic extract induced 76.65% writhing inhibition while 250 and 500 mg/kg b.w. produced 69.57% and 89.96% writhing inhibition, respectively, in the acetic acid test for peripherally mediated analgesia [76,77]. The inhibitory activities of the methanolic leaf extracts against peripheral pain were evidenced to be more effective than the standard, diclofenac sodium, in both studies [76,77]. In a subsequent investigation, Bhowmick and co-workers (2014) [25] showed that 500 mg/kg b.w. of the crude methanolic extract of *L. glutinosa* leaves was the most effective for inhibition of the response against thermal stimuli in the hot plate test (15.54 ± 0.37 s latency) and acetic-acid-induced writhing inhibition (56.32%) compared to the ethyl acetate, n-hexane and chloroform fractions. The study also showed that the dose-dependent analgesic activities of the methanolic extracts was comparable to the standard drug, ketorolac (16.38 ± 0.27 s latency; 64.36% inhibition). In addition to the methanolic extracts, the ethanolic extract obtained from *L. glutinosa* leaves also showed significant antinociceptive properties where 300 mg/kg b.w. extract inhibited 65% of the nociception induced by acetic acid and increased the mean basal latency in tail-flick responses induced by noxious heat [78]. The findings from these studies indicate that the active principles in *L. glutinosa*, especially its leaves, may have acted on opioid receptors as well as inflammatory mediators to achieve successful supraspinal, spinal and peripheral antinociception in all the central (hot plate and tail flick) and peripheral pain models [90,135]. Nevertheless, further mechanistic studies are warranted to confirm the mode of analgesic action of the plant. Additional investigation on the bark of the plant is also necessary to investigate its effectiveness against peripherally induced pain.

### 3.6. Litsea guatemalensis Mez.

*Litsea guatemalensis* Mez. is a shrub or small tree species that is also commonly called ‘*laurel*’, ‘*aguarel*’, ‘*laurelillo*’, ‘*laurel silvestre*’ or ‘*arrayán*’ [26,50,136]. It is a native species from Mexico and Central America that is widely distributed in America as it thrives in mixed or open pine forests [50,136]. The plant is characterized by its long, straight trichomes of variable density, leaves with an acute to attenuate base and a long acuminate apex. The plant is of ornamental value whereas its leaves are widely commercialized as a form of spice in areas where it is commonly found. Moreover, it has been reported to have the widest ethnomedicinal application amongst the *Litsea* species in the region [50]. 

In Mexico, boiled leaf infusions or vapors of the plant are taken as a remedy for fevers, headache, chills, stomachache, diarrhea and emesis or gargled to heal throat infections [45,50]. Pastes made from the ground leaves applied onto the affected site have also been used to treat arthritis and skin conditions [45,136]. Often, baths infused with the leaves were also used to relieve fevers, chills and urinary tract infections as well as broken bones [45]. Other recorded medicinal uses of the plant include treatment for respiratory and gastrointestinal diseases, trauma, muscular pain, rheumatism, stings, cultural affiliated syndromes, colic, swellings and disease of the circulatory and nervous system as well as renal diseases [50,58,136]. The therapeutic uses of *L. guatemalensis* have been attributed to its high composition of linalool, a major monoterpene found in the essential oils of various aromatic plants, as it has remarkable sedative, antinociceptive, anti-inflammatory and antiseptic effects [50].

According to the phytochemical analysis conducted by Vallverdú et al. (2005) [136], 74 compounds were identified in the essential oil of *L. guatemalensis* leaves sampled from Guatemala. Oxygenated monoterpenes, including 1,8-cineole (26.8%), α-terpineol (14.5%), linalool (10.8%) and terpinen-4-ol (6.8%), constituted the majority of the compounds identified (72.2%). Other monoterpene hydrocarbons such as α-pinene (3.7%), limonene (3.6%) and γ-terpinene (2.8%) and sesquiterpenoids such as E-nerolidol (2.5%), β-caryophyllene (1.1%) and β-caryophyllene oxide (0.5%) were also characterized. Meanwhile, another compositional investigation on the *L. guatemalensis* leaf oil from Mexican Bay (San José Yashitinín, Chiapas) revealed the presence of 26 compounds with linalool (21.9%), limonene (16.4%), α-pinene (10.7%) and isobornyl acetate (5.7%) making up the majority of the components in the oil [50]. A subsequent study by Silva et al. (2012) [26] reported the isolation of pinocembrin and scopoletin from the dichloromethane fraction and 5,7,3′,4′-tetrahydroxy-isoflavone from the methanolic fraction of the ethanolic extract via chromatographic methods. In addition to the major compounds, 1,8-cineole and linalool, identified in both the Guatemalan and Mexican varieties of *L. guatemalensis* that have been described earlier to have analgesic potential (refer to the section on *L. glaucescens*), all the three compounds isolated by Silva et al. (2012) [26] have also been determined to possess anti-inflammatory and antinociceptive capacities [26,137,138,139], suggesting that the plant may have useful applications for pain relief. Indeed, the study by Silva and colleagues (2012) [26] successfully demonstrated that the ethanolic extract of *L. guatemalensis* was effective against hyperalgesia. This was in addition to the antimicrobial, anticancer and antioxidant activities that have been reported for the extracts and essential oil of *L. guatemalensis* [50,58,140,141,142,143].

Silva and co-workers (2012) [26] employed the partial sciatic nerve ligation (PSNL) method to determine the effects of the ethanolic extract of *L. guatemalensis* and 5,7,3′,4′-tetrahydroxy-isoflavone on persistent pain following the failure of the extract to show significant inhibition on both phases of formalin-induced nociception. In total, 32% and 41% inhibition of mechanical hyperalgesia was reported for the group that received intraperitoneal treatment with 30 mg/kg b.w. of extract and 1 mg/kg b.w. of the isolated isoflavone, respectively, 4 days post PSNL surgery. The neuropathic pain inducible by PSNL is a form of chronic pain involving complex mechanisms where both the peripheral and central nervous systems may be directly or indirectly implicated [144,145,146]. At the same doses, the extract and isoflavone showed maximum anti-inflammatory activity in the carrageenan-induced paw edema with inhibitions of 62.7% and 62.2%, respectively [26], suggesting that the extract and isoflavone were able to modulate inflammatory mediators such as COXs, prostaglandins, NO and cytokines to reduce carrageenan-induced inflammation [147]. Moreover, the extract and isoflavone were also able to reduce cell migration, predominantly neutrophils, to the pleural cavity [26]. Thus, the findings by Silva et al. (2012) [26] indicated that the anti-hyperalgesic effect of the *L. guatemalensis* leaf extract, largely attributable to its composition of 5,7,3′,4′-tetrahydroxy-isoflavone, was associated with its strong anti-inflammatory effect via the attenuation of lymphocyte influx to the pleural cavity. 

### 3.7. Litsea lancifolia (Roxb.) Hook. F.

*Litsea lancifolia* (Roxb.) Hook. F., name of which is derived from its lance-shaped leaf blades, is a bush or small tree species approximately 8 to 12 m tall [148,149]. The plant is widely distributed, stretching from India to China, and has also been reported as a dominant timber-producing species in the heath forests of South East Asian regions including Sarawak, Sumatra and Singapore [148,150,151]. Locally, it is commonly known as narrow-leaved ‘*medang*’ in Singapore or ‘*judijaylla*’ by the Chakma tribes in Bangladesh [148,152]. It has elliptic acute or acuminate leaves that are pinkish red when young, as well as green ellipsoid apiculately-shaped fruits with white dots that turn dark purplish black upon ripening [149,151]. In addition to being a suitable fuel wood species [153], the roots of *L. lancifolia* have also been traditionally used to treat diarrhea in Bangladesh [152]. On the other hand, the leaves of *Litsea lancifolia* var. *lancifolia* have been reportedly applied in the form of a poultice for healing boils [154].

Several studies have been conducted to determine the phytochemical content of *L. lancifolia*. According to the study by Yang and colleague (2008) [155] from Kunming, China, seven compounds including (−)-aristortetralone, dehydrodiisoeugenol, dihydrodehydrodiconifery alcohol, 5,7-dimethoxy-3’, 4’-methylenedioxyflavan-3-ol, p-hydroxy-benzoic acid, *p*-sitosterol and vanillin were isolated from *L. lanciflora*. In addition, Sulaiman et al. (2011, 2012) [149,156] from Malaysia isolated a total of nine alkaloids including actinodaphnine, boldine, cassythicine, *N*-allyllaurolitsine, Norboldine, *O*-methylarmepavine, pallidine and reticuline, as well as a new bisbenzylisoquinoline, lancifoliaine, from the bark of *L. lancifolia*. Antiplasmodial, antibacterial, hypotensive, antitumor and anti-inflammatory properties have been reported for bisbenzylisoquinoline-type alkaloids [156]. This suggests that the *L. lancifolia* may have potential therapeutic use against malaria, bacterial infections, hypertension, tumors and inflammatory illnesses. Based on pharmacological investigations, the methanolic extract of the leaves of *L. lancifolia* and its various fractions demonstrated notable antioxidant, analgesic, antimicrobial, CNS depressant, hypoglycemic, anti-diarrheal and anti-diabetic activities [20,152,157].

Thus far, only one study has been conducted on the antinociceptive effect of *L. lancifolia*. As part of the screening for pharmacological effects of the *L. lancifolia* leaves, the crude methanolic extract was demonstrated to have significant peripheral analgesic activity [20]. Specifically, 100 and 200 mg/kg b.w. doses of the extract inhibited acetic-acid-induced writhing in the treated mice by 69.45% and 77.96%, respectively. The writhing inhibition caused by the 200 mg/kg b.w. dose was comparable to that induced by the standard drug, indomethacin, at 10 mg/kg b.w. which has shown 79.66% inhibition. As mentioned earlier (refer to the section on *Litsea cubeba*), inflammation plays a significant role in the nociception induced by acetic acid [89]. Since the extract of the leaves of *L. lancifolia* was able to inhibit acetic-acid-induced writhing, it implies that the antinociceptive effect of the plant may occur via attenuation of the synthesis or release of pro-inflammatory factors such as prostaglandins, COXs and cytokines at the peripheral tissues [90]. Nevertheless, further assessment on the central antinociceptive activity of *L. lancifolia* is necessary in order to further determine the mechanism of analgesia of the plant. 

### 3.8. Litsea liyuyingi Liou Ho

*Litsea liyuyingi* is an evergreen shrub species that can be found in the tropics and subtropics of Asia, North and South America and Australia [22]. It has alternating leaves with hairy branchlets and can grow up to 3 m tall [22]. In Bangladesh, it is locally referred to as ‘*Pipulta*’ and has popular use in traditional medicine for the treatment of erectile dysfunction, leucorrhea and gestational diabetes [158,159,160,161]. Traditionally, it has also been used as a tonic and stimulant [158]. Specifically, the leaves and bark of the plant were used as a remedy for leucorrhea in the Langrabazar village of the Bogra district [158], whereas the bark, mixed with the roots of *Ipomoea mauritiana*, bark of *Terminalia arjuna*, bark of *Bombax ceiba*, roots of *Trigonella foenum-graecum* and roots of *Vernonia patula*, is crushed and consumed to treat erectile dysfunction in the villages near the Padma River in the Rajshahi district [160]. To the best of our knowledge, only one study has been conducted to determine the biological activities of *L. liyuyingi*. In the study, the leaf extract and fractions of *L. liyuyingi* demonstrated notable thrombolytic, membrane-stabilizing, antioxidant, antibacterial, antinociceptive, anti-diarrheal and hypoglycemic activities [22].

The in vivo peripheral and central antinociceptive effect of the methanolic extract of *L. liyuyingi* was demonstrated by both the acetic-acid-induced writhing test and the tail immersion test [22]. In the acetic-acid-induced writhing test, 200 and 400 mg/kg b.w. of the extract inhibited the number of writhing actions in the treated mice by 45.73% and 42.83%, respectively, comparable to the standard drug diclofenac sodium which showed 65.71% writhing inhibition. Meanwhile, the same doses of the extract have also significantly increased the latency of the tail withdrawal response of the mice from the hot water source. The maximal effect (5.39 ± 0.77 s latency) was demonstrated by the 200 mg/kg b.w. dose following 90 min treatment time, which was slightly higher compared to the effect of diclofenac sodium (4.73 ± 0.109 s latency). As the *L. liyuyingi* extract has demonstrated significant pain inhibition in both models of peripheral and centrally mediated analgesia, it suggests that the active components of the plant may be able to induce both anti-inflammatory and opioid-like effects to alleviate pain [89,90,135]. Based on the phytochemical screening, Laboni et al. (2017) [22] attributed the analgesic effect of the *L. liyuyingi* leaf extract to its alkaloid content as alkaloids such as morphine, codeine and quinine are known to produce mild to strong analgesic effects [162].

### 3.9. Litsea monopetala Roxb./Litsea polyantha Juss. 

*Litsea monopetala* Roxb., synonymous to *Litsea polyantha* Juss., is a small to medium-sized evergreen tree that grows up to 18 m in height and 60 cm in diameter [81,163]. It is predominantly distributed in Nepal, Bangladesh and Northeast India, but can also be found in Yucatan, Southern Mexico, West Indies, the Bahamas, Bermuda, Florida, Philippine, Sri Lanka, Palestine, South and Central America, China, Burma, West Malaysia, Thailand and Myanmar [23,164]. The plant is characterized by its leaves that are elliptically oblong with rounded tips and pubescent on the underside, ovoid black fruits and flowers that are small and pale yellow, usually occurring in fives or sixes in rounded umbellate heads [81]. Other than being a primary food plant for the cultivation of muga silk worms [165], *L. monopetala* also serves as an economically vital medicinal plant [63].

In folk medicine, the plant is considered to have stimulant, astringent, spasmolytic, antidiarrheal, analgesic, antiseptic, antidepressant, anti-infertility, cytotoxic, antifungal, insecticide, purgative and laxative properties [42,59,60,64]. It has been claimed to have therapeutic effects against pains, bruises, contusions, arthritis, stomach aches, diarrhea, dysentery, diabetes, dislocation, bone fractures, gonorrhea, skin diseases and boil [45,59,60,61,64,65]. In Pakistan and India, the bark has been used as a nerve and bone tonic, analgesic and antiseptic for the treatment of stomachache and arthritis [61]. When made into an aqueous mixture with some sugar, the bark has also been claimed to treat diarrhea and dysentery, whereas pulverized or macerated bark or root could be applied topically to manage pain due to blows, cuts, bruises, contusions, swellings, strenuous work or fractures [60,62,65,81]. Alternatively, the fresh green leaves of the plant were also believed to heal diarrhea as well as dislocation, arthritis and bone fractures [45], although it has also been used as a purgative and laxative [64]. Meanwhile, ointment made from the seed oil has also been used to relieve rheumatism [65]. The extensive use of *L. monopetela* in traditional medicine may be due to the presence of bioactive compounds in various parts of the plant.

*L. monopetela* has been reported to contain alkaloids, butanolides, amides, butenolactones, steroids fatty acid, lignans, terpenes, flavonoids, tannins, saponins and sterols [42,65,80]. Preliminary phytochemical screening on the *L. monopetela* leaves revealed the presence of alkaloids, tannins, saponins, cardiac glycosides and anthraquinone glycoside in the petroleum ether, chloroform and ethyl acetate partitions, but not carbohydrates and reducing sugar [81]. According to other studies, an arabinoxylan comprising a 1:2 molar ratio of d-xylose and l-arabinose was reported in the leaf extract, whereas β-sitosterol, actinodaphnine, eugenol, chalcone, methylchalcone and 1,2-diphenyl-2-butene-1-one, tetradecanal, tridecanol, myristic acid and tridecanal were reported in the bark [81,166]. Decanal, nonanol and capric acid were also identified in fruit oil, whereas the oil of the flower was constituted of α-caryophyllene alcohol, pentacosane, caryophyllene oxide, humulene oxide and tricosane [81]. Hossen and colleagues (2019) [80] suggested that the analgesic, antiemetic and anxiolytic effect observed in the methanolic leaf extract was due to its composition of terpenes, flavonoids, tannins, saponin and sterol. Additionally, the native medicinal use of the plant as an analgesic was associated with its eugenol content which has demonstrated antinociceptive activities [166]. 

Investigation of the analgesic effect of *L. polyantha* (*L. monopetela*) showed that 90% methanolic extract of the bark, at 50, 75 and 100 mg/kg b.w., was effective against acetic-acid-induced nociception, as it reduced 34.2% to 56.5% of writhing frequencies in treated mice [82]. Its effect at the dose of 100 mg/kg b.w. was comparable to the standard drug, aspirin. Manik and colleagues (2010) [82] also revealed that the bark extract exhibited dose-dependent central analgesic activity by the inhibition of nociceptive responses up to 60.4% and 100% in both the tail-flick and hot plate test methods, respectively. Follow-up studies showed that the bark extract was also able to inhibit thermally induced hyperalgesia by up to 67.8% in the tail immersion tests and suggested the possible inhibition of the µ-opioid as the mechanism for its antinociceptive action [65]. As the bark extract could also reduce acetic-acid-induced algesia, it suggests that the active components of the bark may have also acted on the inflammatory pathway via regulation of histamine, serotonin, cytokines and eicosanoids levels to diminish nociception [89].

In comparison, the methanolic extract of the leaves, at the doses of 100, 200 and 500 mg/kg b.w., produced 33.89%, 38.98% and 68.75% writhing inhibition, respectively, following acetic-acid stimulation [23,81]. By comparison, the bark of *L. monopetela* was more effective at alleviating peripheral nociception compared to the leaves, as the bark extract showed a higher inhibitory activity than the leaf extract at the same concentration of 100 mg/kg b.w. in the acetic-acid-induced writhing model for deducing peripheral analgesia. The analgesic effect of the leaves of *L. monopetela* was also assessed using the formalin-induced paw licking test which expresses two phases of nociception—an early non-inflammatory phase defined by the direct influence of formalin on the nociceptors and a late phase that is indicative of inflammatory pain [80]. In this test, 200 and 400 mg/kg b.w. of the methanolic leaf extract of *L. monopetela* significantly reduced the licking time in both phases of pain, whereby the higher dose produced 77.5% and 66.67% inhibition in the early and late phases, respectively [80]. This finding indicated that the leaf of *L. monopetela* has the ability to abate analgesia via the non-inflammatory and inflammatory pathways by desensitizing the sensorial C-fibers and reducing substance P release and modulating the augmented release of inflammatory mediators such as prostaglandins, COXs and NO [90].

## 4. Discussion

Naturally-derived analgesic products have recently grown in popularity as they represent cost-effective alternatives to synthetic ingredients, which are often associated with adverse side effects [17,18]. A number of plants from the *Litsea* genus have been claimed to possess pain-relieving properties according to traditional ethnomedicinal records (Table 1). Based on the literature, nine species from the *Litsea* genus, including *L. cubeba*, *L. elliptibacea*, *L. japonica*, *L. glutinosa*, *L. glaucescens*, *L. guatemalensis*, *L. lancifolia*, *L. liyuyingi* and *L. monopetala*, have been reported to possess potent antinociceptive or relevant activities as revealed via various in vivo, in vitro and clinical studies as shown in Table 2. The reported analgesic activity of these plants emanated from various parts of the plants including fruit, leaves and bark, which have been analyzed in the form of essential oils, extracts or fractions. 

Analgesic compounds generally target, directly or indirectly, nociceptive pathways of the peripheral nervous system, central nervous system or both. Various assays have been adopted by researchers to quantify pain-like behaviors in animal models in an effort to understand the mechanisms involved in the antinociceptive action of *Litsea* plants including the acetic-acid-induced writhing test, tail-flick test and hot plate test. The acetic-acid-induced writhing test is a method that has been used to evaluate the peripheral antinociceptive activities of plant extracts and their natural compound [3]. The method involves the administration of acetic acid at low concentrations into the peritoneal cavity of mice to induce abdominal writhing which is defined by arching of the back, extension of the hind limb and contraction of abdominal musculature [3]. Among the *Litsea* species that have been evaluated for analgesic activities, the fruit oil of *L. cubeba*, ethanolic fruit extract, CH_2_Cl_2_ fraction and major hamabiwalactone constituents of *L. japonica*, methanolic and ethanolic leaf extract of *L. glutinosa* and methanolic leaf extracts of *L. lancifolia*, *L. liyuyingi* and *L. monopetala* have been shown to successfully reduce the frequency of acetic-acid-induced writhing in treated animals [20,21,22,23,24,25,76,77,78,82]. 

The antinociceptive activities of the extracts were deduced by the decrease in frequency of acetic-acid-induced writhing [167]. Writhing is an overt response to the intense pain induced by acetic acid due to the activation of nociceptors in response to the release of chemical and inflammatory mediators such as potassium, H^+^, ATP, bradykinin and PGE_2_ [168]. These substances trigger the liberation of algesic mediators including histamine, 5-HT, nerve growth factors (NGF) and prostanoids from other cells and afferent nerves, which lower pain thresholds and sensitize nociceptive neurons [167,168]. Other studies have also reported that the writhing response is mediated by the acid-sensitive ion channels of adjacent peritoneal mast cells which contribute to the increased nociceptive sensitivity of the neurons [3,169]. In view of the reported efficacies of the abovementioned plant extracts of the *Litsea* species at mitigating acetic-acid-induced writhing, it suggests that the active principles of these *Litsea* extracts were able to reduce peripheral nociception either by decreasing the sensitivity of the ion channels of adjacent peritoneal mast cells to acid, the direct impediment of analgesic mediators or by obstructing the release of peripheral-pain-inducing chemical and inflammatory mediators. Specific interactions with 5-HT_1A_R have been reported for the methanolic bark extract of *L. elliptibacea* and active components of *L. glaucescens*, although the antinociceptive activities of *L. elliptibacea* and *L. glaucescens* have yet to be inspected via conventional pain models [73,75]. Interactions with 5-HT_1A_R have been shown to inhibit nociception following full and partial agonism [94,96,97,98,99,100]. In this regard, *L. elliptibacea* and *L. glaucescens* have the propensity to alleviate nociception by abating the downstream effects of 5-HT to increase pain thresholds and desensitize nociceptive neurons.

Another common model used to assess antinociception involves the thermal induction of analgesia at the central level of the nervous system, such as the tail-flick assay. In this assay, thermal stimulus is focused on the mice’s tail to induce the tail “flick” response and the tail-flick latency is adjudged as an antinociceptive activity [170]. The application of thermal stimulus can be in the form of radiant heat where a high-intensity beam of light is aimed at the rodent’s tail or hot water where the distal end of the tail is submerged into a water bath set at a constant temperature between 46 °C and 52 °C [90]. Similar to the tail-flick assay, the hot plate test also involves the use of thermal stimulus; however, the stimulus is applied to the hind paws of the mice. Furthermore, the reactions observed in the hot plate test are considered as supraspinal-integrated responses rather than spinal responses as in the tail-flick assays [171]. According to the current literature, the fruit oil of *L. cubeba* (at high dose), ethanolic fruit extract, CH_2_Cl_2_ fraction and major hamabiwalactone constituents of *L. japonica*, methanolic and ethanolic leaf extracts of *L. glutinosa* and methanolic extracts of *L. liyuyingi* and *L. monopetala* have demonstrated evident central analgesic activities [22,23,24,25,65,72,78,80,125]. Specifically, the antinociceptive responses induced by the fruit oil of *L. cubeba*, ethanolic fruit extract, CH_2_Cl_2_ fraction and major hamabiwalactone constituents of *L. japonica*, ethanolic leaf extract of *L. glutinosa* and methanolic extracts of *L. liyuyingi* and *L. monopetala* were observed at the spinal level [22,24,65,72,78], whereas for the methanolic leaf and ethanolic bark extracts of *L. glutinosa*, and the methanolic extract of *L. monopetala*, the analgesic reactions were considered supraspinal [25,65,80,125]. 

The delay in the manifestation of nocifensive behaviors following thermal stimulus in both the tail-flick and hot plate assays, described as forepaw withdrawal, hind paw withdrawal, licking, stamping, leaning posture and jumping, were deduced as the antinociceptive activity of the tested extracts [171]. The responses, evoked by a thermal stimulus, are due to the depolarization of C and Aδ nociceptors in the skin’s surface layers as a result of the opening of transient receptor potential vanilloid subtype 1 (TRPV1), an endogenous transducer of noxious heat [172,173]. The activation of TRPV1 allows the entry of Ca^2+^ which promotes the formation and liberation of endogenous mediators such as eicosanoids, acidosis, ATP, histamine, bradykinin and NGF and also vasoactive neuropeptides such as substance P and CGRP [173]. These substances not only further sensitize and activate TRPV1 on the nerve terminals but also induce neurogenic inflammation leading to pain [174,175]. Based on this notion, the antinociceptive effect of the *Litsea* species at the central nervous level may potentially occur via the deactivation of TRPV1 which would lead to downregulation of the endogenous mediators and vasoactive neuropeptides and thus reduce nervous sensitivity and pain-inducing neurogenic inflammation.

In addition to the above assays, PSNL represents another technique that has been used to determine the analgesic profiles of potential pain relievers specifically against persistent neuropathic pain as it is able to simulate some common features of clinical neuropathic pain such as hyperalgesia, dysesthesia and allodynia [144,145]. This assay was adopted to evaluate the pain-relieving effects of *L. guatemalensis* and its isolated compound, 5,7,3′,4′-tetrahydroxy-isoflavone, where treatment with both has been shown to successfully inhibit mechanical hyperalgesia comparable to the effects of gabapentin, a standard positive control used in in vivo persistent pain models [26]. Neuropathic pain has been described to originate and be modulated by both peripheral and central nervous systems [26]. However, the immune system has also been demonstrated to be highly involved in the process [144]. Earlier studies have shown that neuronal damage would prompt a cascade of inflammatory responses including activation of the complement system, recruitment of inflammatory cells to the site of injury, dorsal root ganglia and spinal dorsal horn and the activation of resident satellite cells, astrocytes and microglia. This is followed by the upregulation of cytokines, chemokines and adhesion molecules, which would thus lead to the synthesis and release of analgesic mediators to instigate neuropathic pain [144]. In particular, activation of the extracellular signal-regulated kinase (ERK)/MAP and JNK/MAP kinase pathways in astrocytes in the dorsal horn and the gracile nucleus has been associated with the pathogenesis of neuropathic pain induced by PSNL [176]. Furthermore, nerve injury following PSNL was also reported to upregulate prostaglandin-synthesizing enzyme, COX-2 and thus cause PGE2 overproduction and overexpression of PGE2 receptors in the injured nerves [177]. In the study by Silva and colleague (2012) [26], the extract of *L. guatemalensis* and its isoflavone have shown potent anti-inflammatory activity based on the carrageenan-induced paw edema assay and its capacity to reduce influx of lymphocytes, mainly neutrophils, to the pleural cavity. Although the specific mechanisms involved in the action of *L. guatemalensis* and its isoflavone to ameliorate neuropathic pain have yet to be elucidated, the findings from the study revealed a possible link between the anti-inflammatory and the anti-hyperalgesia effects of the extract and isoflavone.

It must be emphasized that there are limited reports available on the exact antinociceptive mechanisms of *Litsea* plants; nevertheless, a couple of studies have suggested that the plants relieved the pain sensation by targeting the inflammatory mediators and cytokines. Zhao et al. (2020) [21] showed that the fruit oil of *L. cubeba* significantly inhibited the nociception to acetic-acid-induced writhing but failed to delay the manifestation of nocifensive behavior in the hot plate test at the maximum dose of 200 mg/kg b.w. In the same study, treatment with the fruit oil was shown to reduce the levels of proinflammatory cytokines (TNF-α, IL-1β, -6, -8 and -17A) and increase the levels of anti-inflammatory cytokines (IL-10). These results thus led them to deduce that *L. cubeba* fruit oil alleviated pain mainly via the peripheral analgesic route by regulating the production and secretion of inflammatory mediators [21]. Nevertheless, a higher dose of 500 mg/kg b.w. could instigate the central analgesic route as demonstrated in the tail-flick test by Chen et al. (2012) [72], although further studies are necessary to confirm the activity at the supraspinal level. The involvement of the inflammatory pathway was also observed for the analgesic activity of the *L. japonica* ethanolic fruit extract, CH2Cl2 fraction and their main constituents, hamabiwalactone A and B. The extracts and compounds were shown to suppress the production of inflammatory mediators (COX-2/PGE_2_ and NO/iNOS) and cytokines (TNF-α, IL-1 and IL-6), potentiating the involvement of the suppressed JNK/p38 MAP kinase and inhibited nuclear factor-kappa B (NF-κB) signaling pathway [24].

Previous studies have often attributed the antinociceptive activity of *Litsea* plants to the phytochemical constituents present. Indeed, some of the major phytoconstituents detected in the plants have demonstrated analgesic-like activity in vitro and in vivo. For instance, 1,8-cineole, a major phytochemical of *L. glaucescens*, has demonstrated antinociception potentially via a non-opioid anti-inflammatory pathway as described earlier (refer to the section on *L. glaucescens*). Citral, the primary component of *L. cubeba* fruit oil, in separate studies has also been reported to exhibit notable antinociceptive properties. A study evaluating the effect of citral on the transient receptor potential (TRP) ion channels found in dorsal root ganglion neurons showed that citral effectively inhibited TRPV1 currents evoked by a pH 5 solution, and the prolonged inhibitory effects of citral subsequently eliminated the TRPV1 current, thus interrupting nociceptive transmission [178]. Subsequent work by Quintans-Junior et al. (2011) [179] on the antinociceptive and anti-inflammatory responses in rodents proposed that citral inhibits inflammation-induced nociception better than the neurogenic nociception through the suppression of prostaglandin synthesis [179]. Correspondingly, citral was revealed to suppress COX-2 and iNOS expression, via the inhibition of NF-κB activation, and activate peroxisome proliferator-activated receptors (PPAR) α and γ, which play an important role in controlling inflammatory responses [180,181]. These results thereby highlighted the therapeutic potential of citral in managing pain induced by inflammation.

In addition to citral, linalool and pinene, which were among the main the constituents isolated from *L. cubeba* fruit and *L. glaucescens* essential oil, were shown to also downregulate the expression of COX-2 and iNOS [96,182]. Extensive research has been carried out to investigate the antinociceptive activity of linalool extracted from various plants and the potential antinociceptive mechanisms of the compound have been thoroughly discussed in a review by de Cássia da Silveira et al. (2017) [183]. The antinociceptive action induced by linalool was based on its ability to inhibit substance P release, block the neurokinin-1 (NK-1) receptor, reduce peripheral and central nerve excitability and inhibit spinal ERK activation in PSNL [183]. Evaluation of the antinociceptive activity of β-pinene isolated from the essential oil of *Eucalyptus camaldulensis* leaves showed that the compound exerted supraspinal antinociceptive actions in rats and suggested that β-pinene might play a role as a partial agonist through the μ-opioid receptors as it successfully reversed the antinociceptive effect of morphine [184]. The analgesic-like activity of α-pinene from *Juniperus oxycedrus* essential oil has also been reported in a study conducted by Rufino et al. (2014) [185] in which α-pinene was able to prevent IL-1β-induced inflammation. Another study revealed that α-pinene could reduce IL-6 and TNF-α formation in peritoneal macrophages of rats [186], suggesting inflammatory mediators and cytokines as possible targets of α-pinene for the induction of antinociceptive action. Taken together, the downstream mechanistic findings for these compounds that constitute major roles in the *Litsea* species could provide valuable insights for understanding the potential pathways involved in the antinociceptive action of the plants at instigating pain relief.

## 5. Conclusions

Pharmacological evaluations have demonstrated the analgesic potential of nine *Litsea* species including *L. cubeba*, *L. elliptibacea*, *L. japonica*, *L. glutinosa*, *L. glaucescens*, *L. guatemalensis*, *L. lancifolia*, *L. liyuyingi* and *L. monopetala*. Among these species, *L. cubeba*, *L. japonica*, *L. glutinosa*, *L. lancifolia*, *L. liyuyingi* and *L. monopetala* have demonstrated antinociceptive capacity via the peripheral analgesic route. Except for *L. lancifolia*, the other five *Litsea* species have also shown central analgesic activity at either the spinal or supraspinal level. Meanwhile, *L. guatemalensis* was able to improve hyperalgesia induced by PSNL. The pain-relieving actions of the fruit oil of *L. cubeba* as well as the extracts and active constituents of *L. japonica* were proposed to occur via regulation of inflammatory mediators as evidenced by their effects on pro- and anti-inflammatory cytokines and mediators. With regard to *L. elliptibacea* and *L. glaucescens*, the bark extracts and bioactive components were revealed to have active interactions with 5-HT_1A_R and were thus speculated to have analgesic potential by disrupting the pain-stimulating actions of 5-HT to limit pain thresholds and sensitize nociceptors. The reported antinociceptive properties of the major phytoconstituents of the *Litsea* plants further advocated the potent antinociceptive capacities of the plants. Overall, the present literature suggested the potential of *Litsea* plants as prospective candidates for development into effective analgesics that can supplement clinically prescribed drugs or be used as alternative therapies for pain management. 

## Figures and Tables

**Table 2:** Pharmacological findings and phytochemicals reported for the *Litsea* species that have exhibited analgesic/antinociceptive activities.

Species/Vernacular Name	Extract	Analgesic/Antinociceptive Study	Other Related Pharmacological Activities Reported	Phytochemicals Reported in the Study	Reference
*Litsea cubeba* (Lour.) Pers.	Fruit oil	50, 100 and 200 mg/kg of *L. cubeba* fruit oil exerted varying inhibitory effects on acetic-acid-induced torsion but not on thermal stimulation-induced pain.	Anti-inflammatory—symptoms of rheumatoid arthritis ameliorated via regulation of inflammatory cytokines (decreased TNF-α, IL-1β, -6, -8 and -17A levels; increased IL-10 level).	**Monoterpenes**: α-Citral (26.42%); β-Citral (21.94%); α-Pinene; Limonene (12.79%); β-Myrcene; Eucalyptol; Linalool; Geraniol **Sesquiterpenes**: γ-Elemene; *Cis*-nerolidol; Caryophyllene oxide Fatty acids: dodecanoic acid ethyl ester	[21]
	Fruit oil	500 mg/kg of *L. cubeba* fruit oil exhibited antinociceptive activity via prolongation of pain response in tail-flick test (maximum activity at 60 min post-treatment). 100 and 300 mg/kg of fruit essential oil did not show significant effects.	Other neuropharmaological effects—anxiolytic and prolonged pentobarbital-induced sleeping time.	**Major compounds: ** Monoterpenes: α-Citral (geranial) (37.16%); β-Citral (neral) (28.29%); *d*-Limonene (22.90%) **Minor compounds: ** **Monoterpenes**: α-Pinene; Camphene; Sabinene; β-Pinene; β-Myrcene; *p*-Cymene; 1,8-Cineol; Terpinolene; Linalool; Citronellal; α-Terpinyl acetate; Neryl acetate; Geranyl acetate; **Sesquiterpenes**: α-Copaene; β-Caryophyllene; β-Copaene; Elixene; α-Caryophyllene; Caryophyllene oxide; **Other compounds**: 6-Methyl-5-hepten-2-one	[72]
*Litsea elliptibacea* Merr.	Methanolic bark extract	Active against the 5-hydroxytryptamine 1a (5HT1a) CNS receptor (84 ± 1% inhibition), agonism of which inhibits transmission of nociceptive signals and thus induces effective pain relief. Not active against the GABA (GABAB) and dopamine (D2S) receptors.	-	-	[73]
*Litsea japonica* (Thunb.) Jussieu	Fruit extract	Improved MMP-9 levels, joint pain, stiffness and function as shown in a randomized, double-blind, placebo-controlled study.	-	-	[74]
	30% ethanolic fruit extract and CH_2_Cl_2_ fraction	50 and 100 mg/kg by weight of the extract, fraction and active compounds each significantly reduced writhing frequency in a dose dependent manner in the acetic-acid-induced writhing test. The extract, fraction and active compounds also increased the tail-flick latency and latency period in the tail flick and hot plate test.	Suppressed inflammatory mediators, including PGE_2_/COX-2, NO/iNOS and pro-inflammatory cytokines such as IL-1, IL-6 and TNF-α. Inhibited IκB phosphorylation and subsequent nuclear translocation of NF-κB (p65/p50) and suppressed JNK/p38 MAPKs phosphorylation—indicates inhibition of LPS-induced inflammatory responses.	**Butenolactones**: Hamabiwalactone A and B	[24]
*Litsea glaucescens* Kunth	Essential oil	Linalool was reported to interact with 5HT1a receptors which inhibits the transmission of nociceptive signals and thus induces effective pain relief.	Showed antidepressant activity in mice subjected to the forced swimming test (FST).	**Monoterpenes**: Linalool; β-pinene	[75]
*Litsea glutinosa* (Lour.) C.B. Rob.	Methanolic leaf extracts and n-hexane, ethyl acetate and chloroform soluble fractions	500 mg/kg of the crude methanolic extract exerted maximum pain inhibitory activity in both hotplate (15.54 ± 0.37 sec latency) and acetic-acid-induced writhing test (56.32%).	The methanolic extract demonstrated significant thrombolytic, anti-inflammatory and anti-pyretic activities.	-	[25]
	90% methanolic leaf extract	250 mg/kg and 500 mg/kg body weight of the extract produced 69.57% and 86.96% writhing inhibition respectively in the acetic-acid-induced writhing test.	-	-	[76]
	Methanolic leaf extract	100 mg/L showed 76.65% writhing inhibition in the acetic-acid-induced writhing test	The extract showed low antimicrobial activity against *Vibrio mimicus* and good antioxidant activity.	-	[77]
	Ethanolic leaf extract	300 mg/kg inhibited the nociception induced by acetic acid by 65% and showed highly significant anti-nociceptive activity in the tail-flick test.	2000 mg/kg did not show any sign of mortality in the acute toxicity study.	-	[78]
	Ethanolic bark extract	100 and 300 mg/kg of the extract significantly increased the pain threshold in hot plate test.	-	-	[79]
*Litsea guatemalensis* Mez.	50% ethanolic leaf extract	Intraperitoneal treatment with 30 mg/kg of extract and 1 mg/kg of isolated isoflavone exhibited prominent anti-hyperalgesic properties in the partial sciatic nerve ligation (PSNL) test model for persistent pain.	Extract and compound showed potent anti-inflammatory action via paw oedema inhibition and inhibition of lymphocyte (mainly neutrophile) influx to the pleural cavity.	**Flavonoids**: Pinocembrin; **Isoflavones**: 5,7,3′,4′-Tetrahydroxy-isoflavone; **Coumarins**: Scopoletin	[26]
*Litsea lancifolia* (Roxb.) Hook. F.	Methanolic leaf extract and petroleum ether, chloroform and ethyl acetate soluble fractions	100 and 200 mg/kg body weight of the methanolic extract demonstrated significant peripheral analgesic activity with acetic-acid-induced writhing inhibition of 69.45 and 77.96%, respectively.	Ethyl acetate fraction possessed highest total phenolic content and free radical scavenging activity. The fractions showed potential antimicrobial activities against *P. aeruginosa*, *E. coli*, *B. cereus* and *S. paratyphi*. Methanolic extract showed significant hypoglycemic activity. All fractions exhibited CNS depressant activity.	-	[20]
*Litsea liyuyingi* Liou	Methanolic leaf extract and petroleum-ether, carbon tetrachloride and aqueous soluble fractions	400 mg/kg methanol extract demonstrated significant in vivo antinociceptive activity in the acetic-acid-induced writhing and tail immersion test.	Methanol extract and its pet-ether fraction exhibited and good thrombolytic and membrane stabilizing effects. Carbon tetrachloride and aqueous fractions showed good membrane stabilizing effects, significant phenolic content and free radical scavenging activities. Extract and fractions showed inhibitory activities against gram positive and Gram-negative bacteria. Methanol extract possessed significant anti-diarrheal and hypoglycemic activity at 400 mg/kg.	Flavonoids, saponins, alkaloids, phenols and tannins	[22]
*Litsea monopetala* (Roxb.) Pers or *Litsea polyantha* Juss.	Methanolic leaf extract	500 mg/kg extract significantly inhibited acetic-acid-induced writhing by 68.75%.	Extract showed antioxidant activity (IC50 = 223.22 µg/mL). Extract significantly reduced frequency of castor oil-induced diarrhea in mice.	-	[23]
	Methanolic leaf extract	400 mg/kg of extract exhibited 66.67% inhibition of paw licking in mice.	200 mg/kg and 400 mg/kg extract showed dose-dependent and statistically significant antiemetic activity and excellent CNS depressant activity in both elevated plus maze (EPM) and hole board method.	Terpenes, flavonoids, tannins, saponin and sterols	[80]
	Methanolic extract of leaves and petroleum, chloroform and ethyl acetate soluble fractions	100 and 200 mg/kg b.w. of methanolic extract of *L. monopetala* showed significant peripheral analgesic activity with writhing inhibition of 33.89 and 38.98%, respectively.	Petroleum ether fraction showed maximum free radical scavenging activity (IC50 = 59.76 ± 0.71 μg/mL). Fractions showed varying antimicrobial activities. 300 mg/kg/day and 500 mg/kg/day doses of the extract significantly decreased blood glucose level on the 5th and 7th day of treatment. 500 mg/kg dose of extract decreased in locomotion of test animals in CNS depressant activity test.	Alkaloids, tannins, saponins, cardiac glycosides and anthraquinone glycosides	[81]
	90% methanolic extract	50, 75 and 100 mg/kg b.w. of the extract showed significant and dose-dependent central analgesic activity in the tail-flick (22.2–60.4% pain inhibition percentage (PIP)), tail immersion (21.2–67.9% PIP) and hot plate (39.9–100% PIP) tests.	-	Alkaloids and flavonoids	[65]
	90% methanolic extract	50, 75 and 100 mg/kg b.w. of the extract demonstrated dose-dependent anti-nociceptive activity in acetic-acid-induced writhing tests (34.2–56.5% reduction).	-	-	[82]
